# Transcriptomic and metabolomic analysis reveals the difference between large and small flower taxa of *Herba Epimedii* during flavonoid accumulation

**DOI:** 10.1038/s41598-022-06761-z

**Published:** 2022-02-17

**Authors:** Weihan Qin, Yong Yang, Yunhong Wang, Xiaomei Zhang, Xiang Liu

**Affiliations:** Chongqing Academy of Chinese Materia Medica, Chongqing, 400065 China

**Keywords:** Plant sciences, Plant breeding, Secondary metabolism

## Abstract

*Herba Epimedii*, as a traditional Chinese herb, is divided into large and small flower taxa, and can invigorate sexuality and strengthen muscles and bones. *Herba Epimedii* is rich in flavonoids, which largely contribute to its medicinal benefits. In our previous studies, we have found that the flavonoids content was much more in small than large flower taxa. To further identify molecular mechanisms of flavonoids metabolism in *Herba Epimedii*, combined metabolome and transcriptomic analyses were performed to profile leaves and flowers. Association analysis revealed that the expression of genes involved in flavonoid biosynthesis showed significant differences between small and large flower taxa. Eleven flavonols significantly increased in small compared to large flower taxa. Moreover, genes encoding *O*-methyltransferase played crucial roles in flavonoids metabolism by an integrated analysis. Taken together, these data highlight the breeding tendency of small flower taxa to improve the quality of *Herba Epimedii*.

## Introduction

The traditional Chinese medicinal herb, Epimedium (also known as horny goat weed, yin-yang-huo, or barrenwort) is frequently used for the treatment of osteoporosis, sexual dysfunction and cardiovascular disease due to its low-cost and minimal adverse effects^1^. Epimedium is a representative traditional Chinese herb that was recognized for its medical benefits over a thousand years ago, and is widely consumed in oriental countries as a functional food, dietary supplement, or in tea, wines, extracts, tablets or capsules^1^. In Chinese Pharmacopoeia, dried aerial regions of *Epimedium brevicornu, E. sagittatum, E. pubescens, E. wushanense* and *E. koreanum* have been recorded in the Pharmacopoeia of People's Republic of China. More than 270 compounds were identified from 52 species, with prenylflavonoids noted as the major components in addition to distinct chemotaxonomic markers in *Epimedium*^1^. Epimedin A, epimedin B, epimedin C and icariin comprise more than 52% of the flavonoids in *Herba Epimedii*, and have been reported for their immunomodulation, anti-osteoporosis and anti-tumor activity^2^.

The biosynthesis of flavonoids is well-characterized in medicinal plants such as *Gnetum parvifolium*^3^, *Chrysanthemum morifolium*^4^, *Lotus japonicus*^5^, and *Anoectochilus roxburghii*^6^. Biosynthesis can be divided into phenylpropanoid and flavonoid pathways. Phenylalanine ammonia-lyase (PAL) is the first enzyme of the phenylpropanoid pathway that converts phenylalanine into cinnamic acid^7^. Cinnamic acid is then converted into p-coumaric acid by trans-cinnamate 4-hydroxylase (C4H). Next, 4-coumarate CoA ligase (4CL) converts coumaric acid into its CoA ester. A key branch point in this pathway is 4CL, as its products are used by various oxygenases, reductases, and transferases for the biosynthesis of lignin, flavonoids, anthocyanins, aurones, stilbenes, coumarins, suberin, cutin and sporopollenin^8^. Chalcone synthase (CHS) and chalcone isomerase (CHI) mediate a two-step condensation reaction, producing naringenin chalcone and naringenin, respectively. Flavanone production is then catalyzed by flavonoid 30-hydroxylase (F30H) and other enzymes. Subsequently, flavanone produces the branches of flavone and dihydroflavonol under the catalysis of flavone synthase (FNS) and flavanone 3-hydroxylase (F3H), respectively. Next, flavonol synthase (FLS) catalyzes C-3 hydroxylation in the dihydroflavonols to produce various flavonols and flavonol-glycosides, formed by flavonoid 3-O-glucosyltransferase (GT) and rhamnosyltransferase (RT) or GT.

*Herba epimedium* is native to China and can be divided into large- and small-flowered groups. These groups are morphologically similar, but show variable flower sizes. The flowers of *E. brevicornu* and *E. sagittatum* are white or pale yellow, with lengths of 1 cm and 0.8 cm, respectively. *Epimedium grandiflorum* is characterized by its larger flowers, measuring 2–4.5 cm. In our previous study, we revealed significant differences in the flavonoid content of the two populations, in which small flowers exceeded those of large flowers. It is of great significance to explore the differential flavonoid synthesis pathways that exist between these flower groups to improve future flavanone-rich cultivation resources of *Epimedium*.

With the rapid development of high-throughput sequencing technologies and systems biology, multi-omics has emerged as an indispensable research method^9,10^. Omics can provide information on dynamic changes in plant growth and development at the systems and cellular level. The metabolome is a powerful approach to qualitatively and quantitatively analyze small-molecule metabolites (mass ≤ 1000 Da) in cells or tissues of an organism during any physiological period. Commonly used techniques include nuclear magnetic resonance (NMR) spectroscopy, liquid chromatography-mass spectrometry (LC–MS), and gas chromatography-mass spectrometry (GC–MS)^11^. These methods can reveal important information on global metabolic changes. Similarly, transcriptomics permits the detection of all RNA transcripts in a sample that reflect changes in gene expression between different samples or treatments^12^. Integrated transcriptomic and metabolomic analyses can be applied to study the metabolic pathways of substances^13,14^, the color formation of vegetables, fruits, and flowers^15,16^, stress resistance mechanisms^17,18^, and the growth and development mechanisms of plants^19,20^. The combination of these approaches can not only be used to elucidate changes in the content of metabolites, but holds utility for the analysis of differentially expressed genes (DEGs).

In this study, transcriptomic databases of *Epimedium* organs were constructed for large and small flowers groups using sequencing platforms and bioinformatic analysis. The results showed that the accumulation of flavonoids in small flowers groups was mainly due to the high levels of O-methyltransferase expression. Flavonoid content and gene expression related to the biosynthesis of flavonoids were further investigated between large and small flowers using UPLC-Q-TOF and qRT-PCR. The data obtained provide important information reference information for future studies on the accumulation and metabolic regulation of flavonoids in *Epimedium*, and can assist the identification of flower varieties with high medicinal value.

## Materials and methods

### Sample collection

Samples were collected from Chognqing, Hunan Province, Sichuan Province, Guizhou Province, China, and identified as large or small flower taxa of *Herba Epimedii*. Flowers and leaves were collected, immediately immersed in liquid nitrogen and stored at − 80 °C prior to transcriptomic and metabolomic analyses. Flowers derived from six cultivars were used for transcriptomic and metabolomic analysis, including three large and three small flower taxa cultivars. Leaves derived from four cultivars were used for transcriptomic and metabolomic analysis, including two large and two small flower taxa cultivars. Details of all samples are shown in Supplementary Table S4.

### Sample extraction and metabolome analysis

Flowers and leaves were freeze-dried and crushed for 1.5 min at 30 Hz using a mixer mill (MM400, Retsch, Germany). Subsequently, 100 mg powder was weighed and extracted overnight at 4 °C in 1.0 mL of 70% methanol. During this period, samples were vortexed (10 s, 40 Hz) once every 10 min on a total of three occasions. Following centrifugation at 10,000 *g* for 10 min at 4 °C, extracts were filtered through a 0.22 µm microporous membrane and stored in sample vials. The QC was prepared by mixing all samples. To examine the reproducibility of the analysis process, a QC sample was injected after five test samples during instrumental analysis.

Sample extracts were analyzed using a UPLC-ESI–MS/MS system (HPLC, Shim-pack UFLC SHIMADZU CBM30A system; MS, Applied Biosystems 4500 Q TRAP). UPLC separation was completed on a Waters Acquity UPLC HSS T3 C18 column (1.8 µm, 2.1 mm × 100 mm) (Waters Corp., Milford, MA, USA). The solvent system consisted of water containing 0.04% acetic acid and acetonitrile containing 0.04% acetic acid. The gradient program was as follows: 100:0 V/V at 0 min, 5:95 V/V at 11.0 min, 5:95 V/V at 12.0 min, 95:5 V/V at 12.1 min, 95:5 V/V at 15.0 min. The flow rate was 0.4 mL/min and the injection volume was 5 µL. The column temperature was set to 40 °C. Effluents were alternatively connected to an ESI-triple quadrupole-linear ion trap (Q TRAP)-MS. Linear ion trap (LIT) and triple quadrupole (QQQ) scans were performed using QTRAP. ESI source operation parameters were as follows: ESI temperature: 550 ◦C; mass spectrometry voltage: 5500 V, ion source gas I (GSI), gas II (GSII), curtain gas (CUR): 55, 60, and 25.0 psi, respectively. QQQ scans were obtained as MRM experiments with collision gas (nitrogen) set to 5 psi. DP and CE for individual MRM transitions were optimized in the QQQ. A specific set of MRM transitions were monitored during each period according to the metabolites eluted within this period.

Based on public databases of metabolites and the MVDB V2.0 database of Wuhan Metware Biotechnology Co., Ltd. (Wuhan, China), qualitative analysis of primary and secondary mass spectrometry data were performed by referencing existing mass spectrometry databases such as MassBank, KNAPSAcK, HMDB and METLIN. Structural analysis of the metabolites was therefore determined. Regarding the quantitative analysis of the metabolites, MRM was used and PCA and OPLS-DA were performed to identify differential metabolites. We divided cultivars into large and small flower taxa group, and then conducted a comparative analysis of large and small flower taxa group. A VIP > 1 and |Log2 (small flower taxa group/large flower taxa group) |≥ 1 were set for metabolites with significant differences.

### RNA isolation and transcriptomics

Total RNA was isolated and purified from leaves and flowers of *Herba Epimedii* using M5 Plant RNeasy Complex Mini Kits (Mei5bio, Beijing, China) following the manufacturer’s recommendations. Samples were treated with an RNase-free DNase I digestion kits (Aidlab, Beijing, China) to remove contaminated genomic DNA. RNA degradation was measured using 1% agarose gels. RNA concentrations were measured on a NanoDrop 2000 spectrophotometer (Thermo Scientific, Wilmington, DE, USA).

Constructed libraries were sequenced using an Illumina HiSeq™ 2000 sequencer (Illumina, San Diego, CA, USA) after quality-checking on an Agilent 2100 Bioanalyzer (Agilent, Palo Alto, CA, USA). Raw data was filtered through the removal of reads with adapter sequences and low-quality reads using fastp software [50]. After filtering, clean reads were assembled to obtain unigenes. Unigene functions were annotated based on NCBI non-redundant protein sequences (Nr, https://ftp.ncbi.nlm.nih.gov/blast/db/FASTA/), KEGG (https://www.genome.jp/kegg), Clusters of Orthologous Groups of proteins (COG, https://www.ncbi.nlm.nih.gov/COG/), GO (https://www.geneontology.org), the Swiss-Prot (http://www.ebi.ac.uk/uniprot/) and the translation of EMBL (TrEMBL) databases. Similarly, we conducted a comparative analysis of large and small flower taxa group. Differentially expressed genes (DEGs) were screened using the following criteria: fold change (Log2) > 1 and P < 0.05. DEGs were subjected to Gene Ontology (GO) (http:// geneontology.org/) and Kyoto Encyclopedia of Genes and Genomes (KEGG) (http://www.genome.jp/kegg/) enrichment analyses to determine their biological functions and metabolic pathways^21–23^.

### Integrative metabolomic and transcriptomic analysis

Transcriptomic and metabolomic data for *Herba Epimedii* flowers and leaves were analyzed. Metabolite content of the different flower taxon and gene expression values in the transcriptomic datasets were analyzed. DEGs in flavonoid biosynthesis and differentially abundant flavonoids in each comparison group (three replicates each) were subjected to correlation analysis. Spearman’s correlation coefficients were used to determine differences between flavonoid biosynthesis genes and transcription factor-encoded genes through the selection of genes with correlation coefficients ≥ 0.90 and correlation p-values ≤ 0.05. O-methyltransferase encoding genes related to flavonoid biosynthesis were selected for analysis.

### Determination of the flavonoid content in the different flower taxon

To validate the differences in flavonoids between large and small flower taxa, 16 monomer flavonoids were tested. Standards of icariin (489-32-7), icariside I (56725-99-6), icariside II (113558-15-9), epimedin A (110623-72-8), epimedin B (110623-73-9), epimedin C (110642-44-9), sagittatoside A (118525-35-2), sagittatoside B (118525-36-3), 2'-*O*-rhamnosyl-icariside II (135293-13-9), baohuoside II (55395-07-8), epimedoside A (39012-04-9), desmethylicaritin (28610-31-3), anhydrocicaritin (116554-17-5), maohuoside (20872-01-4), baohuoside V (118544-18-6), acuminatin (143601-07-4) were purchased from Alfa Biological Technologies (Chengdu, China). Sample purity was confirmed as ≥ 98%. Standard samples were dissolved in methanol to obtain 1 µg/mL standard working solutions. Samples were stored at − 20 °C and used within one month.

Sample preparation (~ 100 mg) was performed as described. Extracts of sample solutions were passed through a 0.22 µm syringe nylon filter and stored at − 20 °C prior to analysis.

HPLC separation was performed using an LC-30A Ultra-performance Liquid Chromatograph System (Shimazu, Japan). The mobile phase consisted of solvent A (water containing 0.02% formic acid) and solvent B (acetonitrile). The gradient procedure was as follows: 0–1.5 min, 8% B, 1.5–9.5 min, 8% ~ 65% B, 9.5–15.0 min, 65%-85% B, 15.0–18.5 min, 85% B, 18.5–18.6 min, 85%-8% B and 18.6–20.0 min, 8% B. The flow rate was 0.2 mL/min and the injection volume was 2 µL.

An Applied Biosystems TripleTOF4600 MS/MS spectrometer equipped with a version of 1.6 Analyst software (AB SCIEX, Massachusetts, USA) was used for analysis. Mass spectrometry was performed on an electrospray ion source using the following parameters: positive ion mode (ESI +); positive ion scanning and IDA (information association acquisition); ion source spray voltage, 5500 V; Ion source temperature, 500; Curtain gas (CUR), 164 kPa; Atomized gas (GS1), 355 kPa; Auxiliary gas (GS2), 355 kPa; Cluster cracking voltage, 55 V; Collision energy, 40 V; Collision energy rolling range 15 V. Scanning range *m/z* 50–1000 compounds with monitoring response values over 1000 cps were set to obtain daughter ion information.

### qRT-PCR analysis

Six unigenes related to flavonoid biosynthesis were selected for validation using qRT-PCR. RNA was reverse-transcribed to cDNA using PrimeScript^TM^ RT reagent Kits (TaKaRa, Dalian, China). qRT-PCRs were performed using the SYBRfiPremix ExTaq TM (TaKaRa, Dalian, China) on a Stepone Real-Time PCR System (ABI, USA). qRT-PCR specific primers were designed using Beacon Designer 8.0 with sequences listed in Table S5. Thermocycling conditions were follows: 95 °C for 5 min; 40 cycles of 30 s at 95 °C, 30 s at 59 °C, and 30 s at 72 °C. The dissolution curve program was used to determine reaction specificity as follows: 60 °C for 15 s followed by 95 °C for 15 s. Relative gene expression was calculated according to the 2^−ΔΔCT^ method. Three biological replicates were analyzed for each gene assayed^24^.

### Statistical analysis

All transcriptome and metabolome samples were assessed from three biological replicates. Statistical analyses were performed using SPSS 22.0 software (IBM, Chicago, IL, USA). An independent samples t-test was used for statistical comparison of large and small flower taxa groups.

### Ethics approval

Collection of plant material in this study complies with institutional, national, or international guidelines.

## Results

### Differentially accumulated flavonoids in *Epimedium* flowers

**Figure 1:**
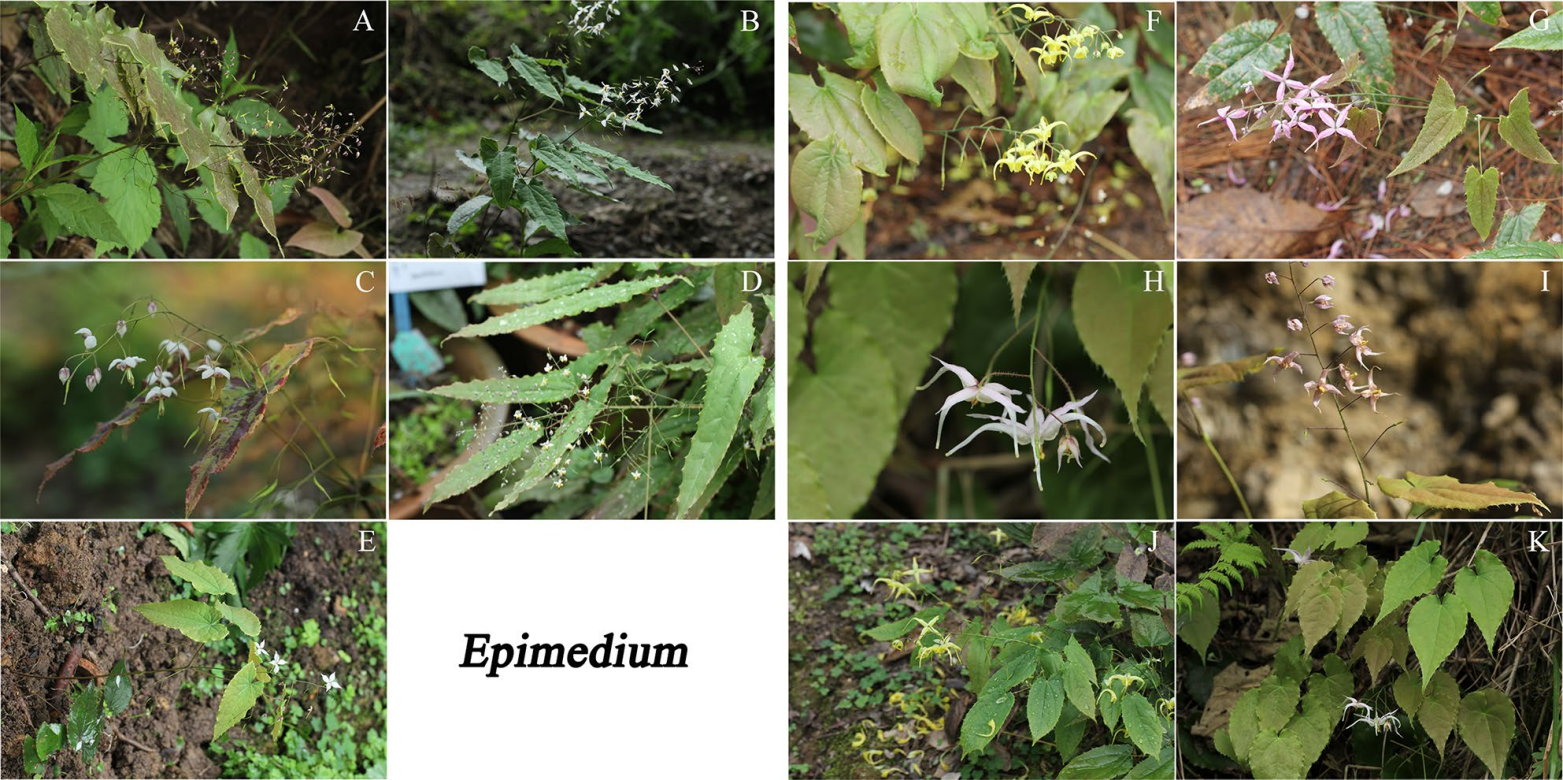
The *Herba Epimedii* cultivars, including five small (**A–E**) and six large (**F–K**) flower taxa.

A total of 11 *Herba Epimedii* cultivars were investigated, including five small flower taxa cultivars (Fig. 1A–E) and six large flower taxa cultivars (Fig. 1F–K). We identified 16 major flavonoids of leaves in the eight cultivars, including four large flower taxa cultivars that included *Epimedium glandulosopilosum* H.R. Liang (S1), *Epimedium leptorrhizum* Steam (S2), *Epimedium sutchuenense* Franch (S3), *Epimedium franchetii* Steam (S4), and four small flower taxa cultivars, including *Epimedium dewuense* S. Z. He (S5), *Epimedium borealiguizhouense* S. Z. He et Y. K. Ying (S6), *Epimedium dolichostemon* Stearn (S7), and *Epimedium myrianthum* Stearn (S8). We assayed the total flavonoid content of leaves in four large and four small flower taxa cultivars. As shown in Supplementary Fig. S1, the total flavonoid content was remarkably higher in small vs large flowers.

### Identification of metabolites

**Figure 2:**
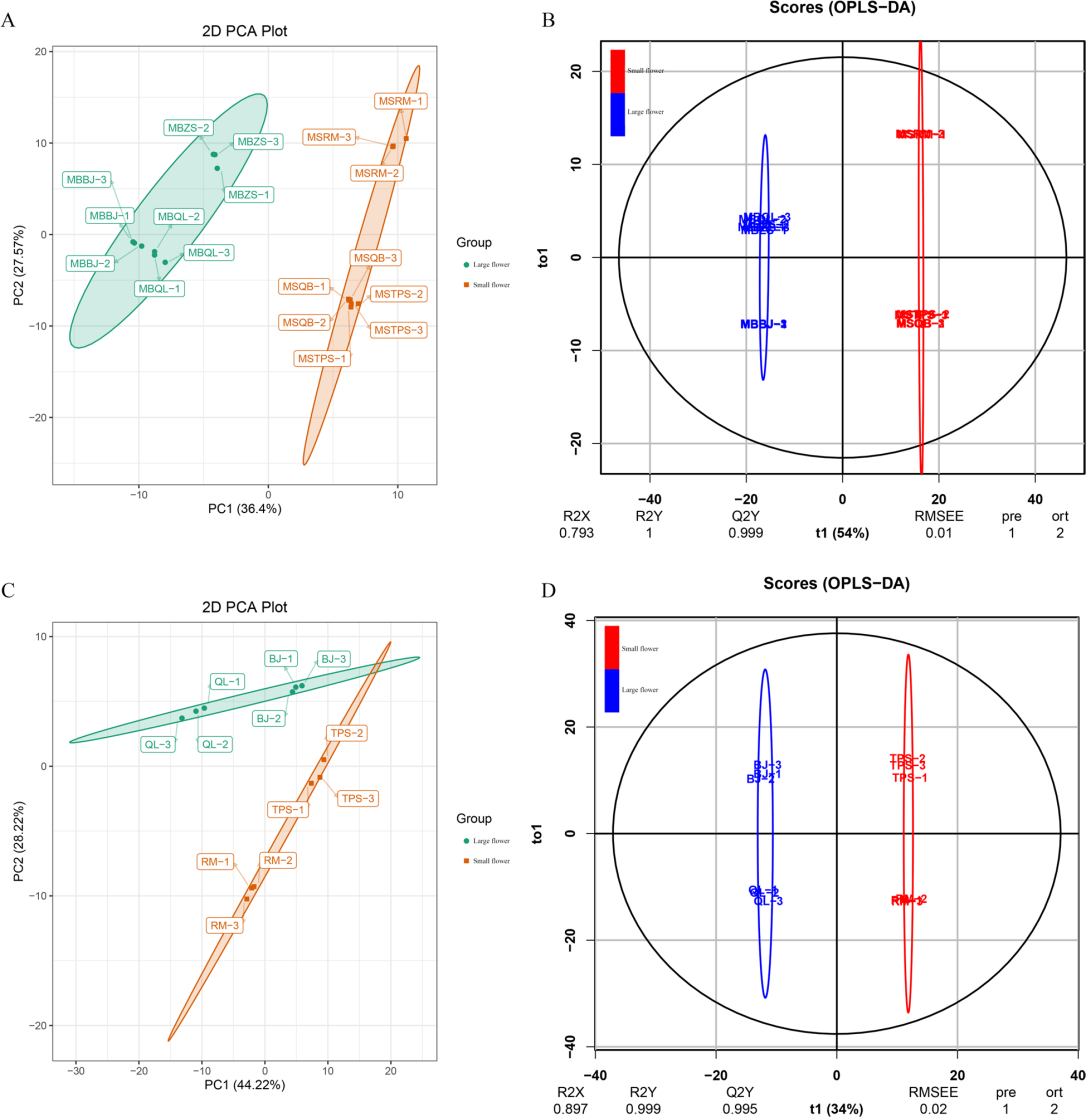
PCA and OPLS-DA scores plots derived from ultra-performance liquid chromatography-electrospray ionization-tandem mass spectrometry (UPLC-ESI–MS/MS) profiling of flowers and leaves in *Herba Epimedii*. **(A)** In flowers, PCA scores plot of the two samples (large and small flower taxa); the x-axis represents the PC1 and the y-axis represents PC2. (**B)** In flowers, OPLS-DA scores plot of the putatively annotated metabolites from large and small flower taxa. The x-axis represents the score value of main components in the orthogonal signal correction process and the differences between the groups can be seen from the direction of the x-axis; the y-axis represents the scores of orthogonal components in the orthogonal signal correction process and the differences within the groups can be seen from the direction of the y-axis. (**C)** PCA scores plot in leaves. (**D)** OPLS-DA scores plot in flowers. All samples information is listed in Supplementary Table S4.

Large and small flower taxa were next assessed through metabolomic analysis in the flowers and leaves. Overlapping analysis of total ion chromatography (TIC) in different quality control (QC) samples showed consistent retention times and peak intensities (Supplementary Fig. S2), indicating high stability of the instrument, permitting its use for subsequent analysis. Principal component analysis (PCA) was performed on large and small flower taxa in the flowers and leaves. Principal component 1 (PC1) and principal component 2 (PC2) were 36.40% and 27.57% in flowers (Fig. 2A), and 44.22% and 28.22% in leaves, respectively (Fig. 2B). The metabolite profiles of *Herba Epimedii* were then subjected to orthogonal partial least squares discriminant analysis (OPLS-DA). The results showed that R2X, R2Y, and Q2 were 0.793, 1.000, and 0.999 in flowers (Fig. 2C), and 0.897, 0.999, and 0.995 in leaves, respectively (Fig. 2D), indicating stability and reliability of the OPLS-DA model. Score plots of PCA and OPLS-DA exhibited a remarkable separation between large and small flower taxa, each forming individual clusters. This highlighted that the flower taxa differentially influence metabolism in *Herba Epimedii*.

**Figure 3:**
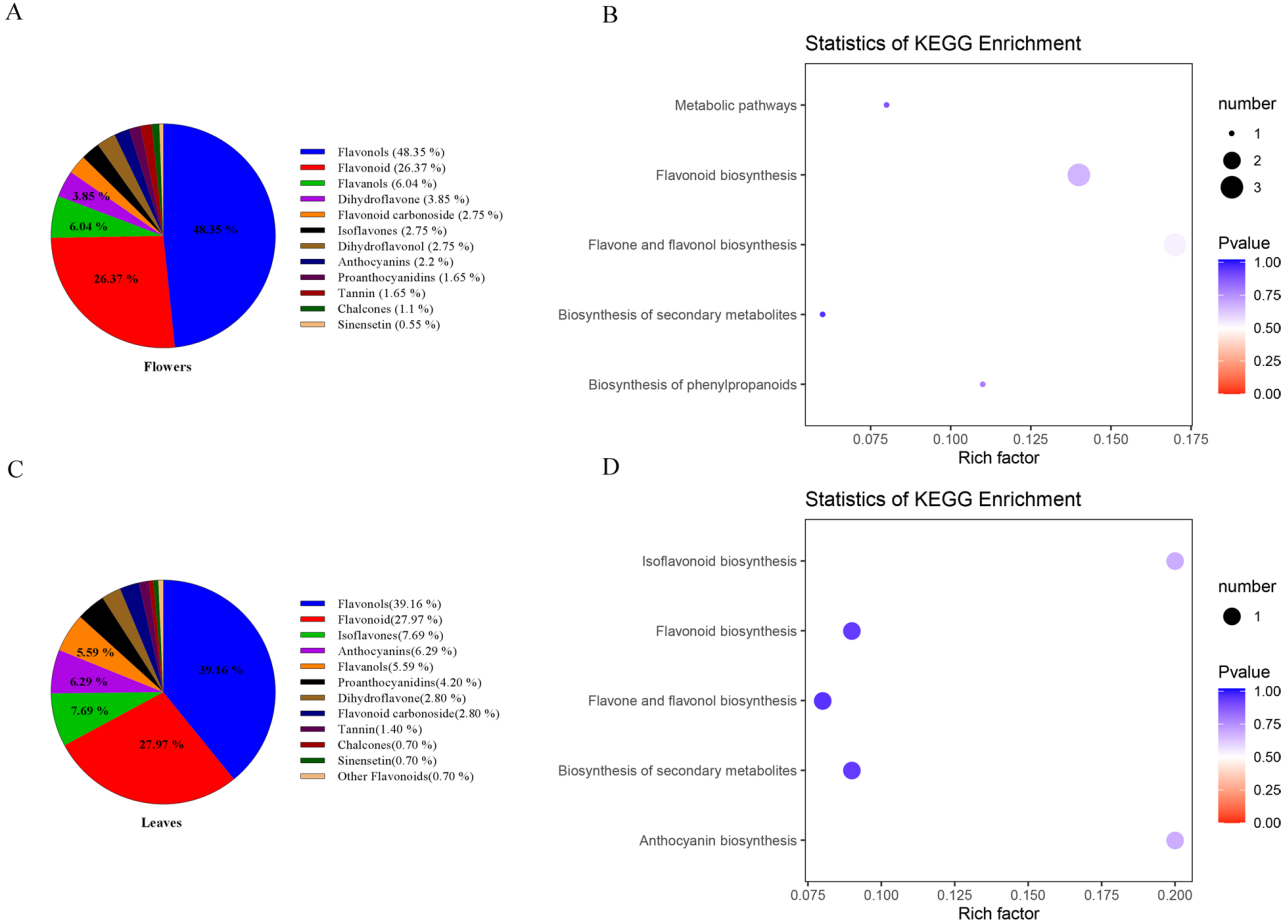
Component analysis of the putatively annotated metabolites and pathway enrichment analysis of the DAMs. **(A)** In flowers, component analysis of the putatively an-notated metabolites from large flower taxa and small flower taxa. The percentage of the top six metabolites are shown in the graph. The percentage after each compound represents the percentage of the number of DAMs of a certain class of compounds in the total DAMs. (**B)** In flowers, metabolic pathway of flavonoids enrichment analysis of the DAMs between large flower taxa and small flower taxa. The x-axis represents the corresponding rich factor of each pathway. The y-axis represents the name of pathway. The color of the dot is p-value, and the closer it is to 0, the more significant the enrichment is. The size of the point represents the number of DAMs enriched in the corresponding pathway. The rich factor is the ratio of the number of metabolites in the corresponding pathway to the total number of metabolites detected and annotated in the pathway. The higher the value of rich factor is, the higher the enrichment degree is. (**C)** In leaves, component analysis of the putatively annotated metabolites from large flower taxa and small flower taxa. (**D)** In leaves, metabolic pathway of flavonoids enrichment analysis of the DAMs between large flower taxa and small flower taxa.

A total of 182 and 143 metabolites with known structures were selected between large and small flower taxa under quality validation in the flowers and leaves (three biological replicates of each). Detailed information on the identified metabolites, including compounds, classes, molecular weights, ionization models, Kyoto encyclopedia of genes and genomes (KEGG) pathways are shown in Supplementary Table S1. In flowers, flavonols (48.35%), flavonoids (26.37%), Flavanols (6.04%) and dihydroflavone (3.85%) accounted for a large proportion of these 182 metabolites (Fig. 3A). In leaves, flavonols (39.16%), flavonoids (27.97%), isoflavones (7.69%) anthocyanins (6.29%) and flavanols (5.59%) accounted for the main proportion of the 143 metabolites (Fig. 3C).

### Identification of differentially accumulated metabolites in flavonoids

Differentially accumulated metabolites (DAMs) were defined as those exhibiting a fold change ≥ 2 or ≤ 0.5 and a variable importance of projection (VIP) ≥ 1. To further understand the function of flavonoid DAMs and their related biological processes, pathway enrichment analysis was performed using KEGG. Interestingly, all flavonoid pathways were not significantly enriched in flowers and leaves, whilst DAMs were specifically enriched in flavonoid pathways in large and small flower taxa (Fig. 3B,D). These data further indicated that flavonoid metabolism in *Herba Epimedii* was significantly influenced by the flower taxa.

A total of 50 and 39 DAMs involved in flavonoid metabolism were identified in flowers and leaves, respectively. In flowers, 43 DAMs were upregulated and 7 were downregulated. Flavonal DAMs accounted for the majority of those upregulated in the small flower taxa, when compared with the large flower taxa (Supplementary Table S2). In leaves, 10 DAMs were upregulated and 29 were downregulated. Flavonol DAMs accounted for a large proportion of the upregulated DAMs (~ 90%), including Maohuoside B, Lcariside II, Epimedin B, Isorhamnetin 3-*O*-β-(2''-*O*-acetyl-β-d-glucuronide), Lcariside I, Epimedoside E, Kaempferol 3-*O*-(6′′-*O*-acetyl)-glucoside, Maohuoside A and Diphylloside B.

### Transcriptomic profiles of large and small flower taxa *Herba Epimedii*

The expression profiles of large and small flower taxa were analyzed using RNA-sequencing (RNA-seq) in flowers and leaves. Q20 and Q30 base percentages were greater than- or equal to 97.42% and 92.61% in leaves (Table 1a) and flowers (Table 1b), respectively. The GC content of the large and small flower taxa were in the range of 45.71–46.32% and 43.94–44.23% in leaves (Table 1a), compared to 43.71–44.76% and 43.94–44.23% in flowers, respectively (Table 1b). Compared to large flower taxa groups, 18,505 and 36,293 genes were differentially expressed in leaves and flowers, including 6823 and 20,730 upregulated genes and 11,682 and 15,563 downregulated genes, respectively.

**Table 1:** Summary of the analysis of transcriptome sequences.

Sample	Raw reads	Clean reads	Clean base (G)	Error rate (%)	Q20 (%)	Q30 (%)	GC content (%)
**(a) Between large flower taxa and small flower taxa in leaves**							
BJ-1	52,812,206	52,417,392	7.86	0.02	97.73	93.38	45.81
BJ-2	54,001,300	53,544,250	8.03	0.02	97.67	93.25	45.94
BJ-3	54,591,534	54,274,534	8.14	0.02	97.54	92.99	45.93
QL-1	55,651,348	55,116,832	8.27	0.02	97.69	93.32	46.32
QL-2	62,712,782	62,340,640	9.35	0.02	97.77	93.5	46.00
QL-3	49,489,270	49,222,574	7.38	0.02	97.42	92.61	45.71
RM-1	49,750,718	49,381,854	7.41	0.02	97.79	93.53	45.27
RM-2	61,365,616	60,901,122	9.14	0.02	97.58	93.05	45.20
RM-3	45,690,070	44,431,084	6.66	0.02	97.88	93.75	45.21
TPS-1	58,406,018	58,058,712	8.71	0.02	97.58	93.06	45.29
TPS-2	52,744,426	52,386,186	7.86	0.02	97.72	93.4	45.36
TPS-3	56,175,820	55,666,340	8.35	0.02	97.64	93.19	45.08

**(b) Between large flower taxa and small flower taxa in flowers**							
TBBJ-1	47,350,042	45,350,814	6.8	0.03	97.64	93.14	44.12
TBBJ-2	47,240,022	45,362,604	6.8	0.03	97.71	93.33	44.14
TBBJ-3	44,670,570	42,890,336	6.43	0.03	97.7	93.27	44.18
TBQL-1	50,597,948	48,290,146	7.24	0.03	97.68	93.29	43.71
TBQL-2	44,880,380	42,930,520	6.44	0.03	97.76	93.44	43.84
TBQL-3	50,740,328	47,948,600	7.19	0.03	97.62	93.13	43.78
TBZS-1	46,056,604	44,620,486	6.69	0.03	97.86	93.62	44.76
TBZS-2	44,127,286	42,693,578	6.4	0.03	97.8	93.53	44.74
TBZS-3	44,329,146	42,511,938	6.38	0.03	97.83	93.58	44.70
TSQB-1	52,454,416	50,026,978	7.5	0.03	97.73	93.39	44.23
TSQB-2	51,593,260	48,730,502	7.31	0.03	97.75	93.37	44.23
TSQB-3	45,734,560	44,212,110	6.63	0.03	97.74	93.36	44.21
TSRM-1	56,835,454	54,524,690	8.18	0.03	97.76	93.44	43.94
TSRM-2	54,811,152	52,412,630	7.86	0.03	97.57	93	44.01
TSRM-3	46,925,704	45,054,414	6.76	0.03	97.48	92.88	43.96
TSTPS-1	54,166,752	51,708,482	7.76	0.03	97.65	93.15	44.15
TSTPS-2	46,303,816	44,241,178	6.64	0.03	97.89	93.73	44.16
TSTPS-3	48,498,632	46,301,992	6.95	0.03	97.64	93.22	44.14

(a) Large flower taxa: BJ and QL; small flower taxa: RM and TPS, all samples information is listed in Supplementary Table S4.

(b) Large flower taxa: TBBJ, TBQL and TBZS; small flower taxa: TSQB, TSRM and TSTPS, all samples information is listed in Supplementary Table S4.

**Figure 4:**
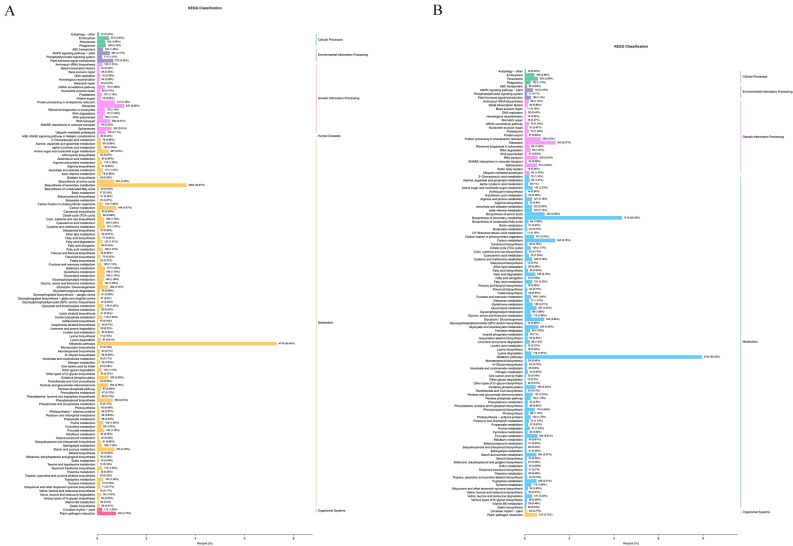
The classification column of KEGG from the DEGs between large and small flower taxa. The x-axis represents the proportion of genes annotated to the pathway in the total of annotated genes. The y-axis represents the name of KEGG pathway. The labels represent the classification of KEGG pathway. (**A)** In flowers. (**B)** In leaves.

Genes with |log2 (fold change)|> 1 and p < 0.05 were defined as differentially expressed genes (DEGs). To further understand the functions of the DEGs and their related biological processes, gene ontology (GO) and KEGG analyses were performed. GO analysis classified the DEGs into three categories: “molecular functions”, “cellular components”, and “biological processes” with a total of 226 and 356 GO terms in flowers and leaves. In flowers, enriched GO terms included “xyloglucan: xyloglucosyl transferase activity”, “anthocyanidin 3-*O*-glucosyltransferase activity”, “ferric iron binding” and “ribonuclease T2 activity” within molecular function, “nuclear nucleosome”, “lipid droplet”, “primary cell wall” and “central vacuole” within cellular component and “xyloglucan metabolic process”, “hemicellulose metabolic process”, and “fatty acid derivative metabolic process” within biological process (Supplementary Table S3). In leaves, “exopeptidase activity”, “oxidoreductase activity, acting on paired donors, with incorporation or reduction of molecular oxygen, NAD(P)H as one donor, and incorporation of one atom of oxygen”, “carboxylic ester hydrolase activity” and “*O*-methyltransferase activity” within molecular function, “plant-type vacuole”, “plant-type vacuole membrane” and “mRNA cap binding complex” within cellular components and “lipid catabolic processes”, “pigment biosynthetic processes” and “auxin metabolic processes” within biological processes (Supplementary Table S3). Pathway enrichment analysis of the DEGs using KEGG identified significantly enriched “metabolic pathways” and “biosynthesis of secondary metabolites” in flowers and leaves (Fig. 4A,B). These transcriptomic analyses confirmed that the flower taxa significantly influenced the metabolic pathways of *Herba Epimedii*.

### Association analysis of the DAMs and DEGs

**Figure 5:**
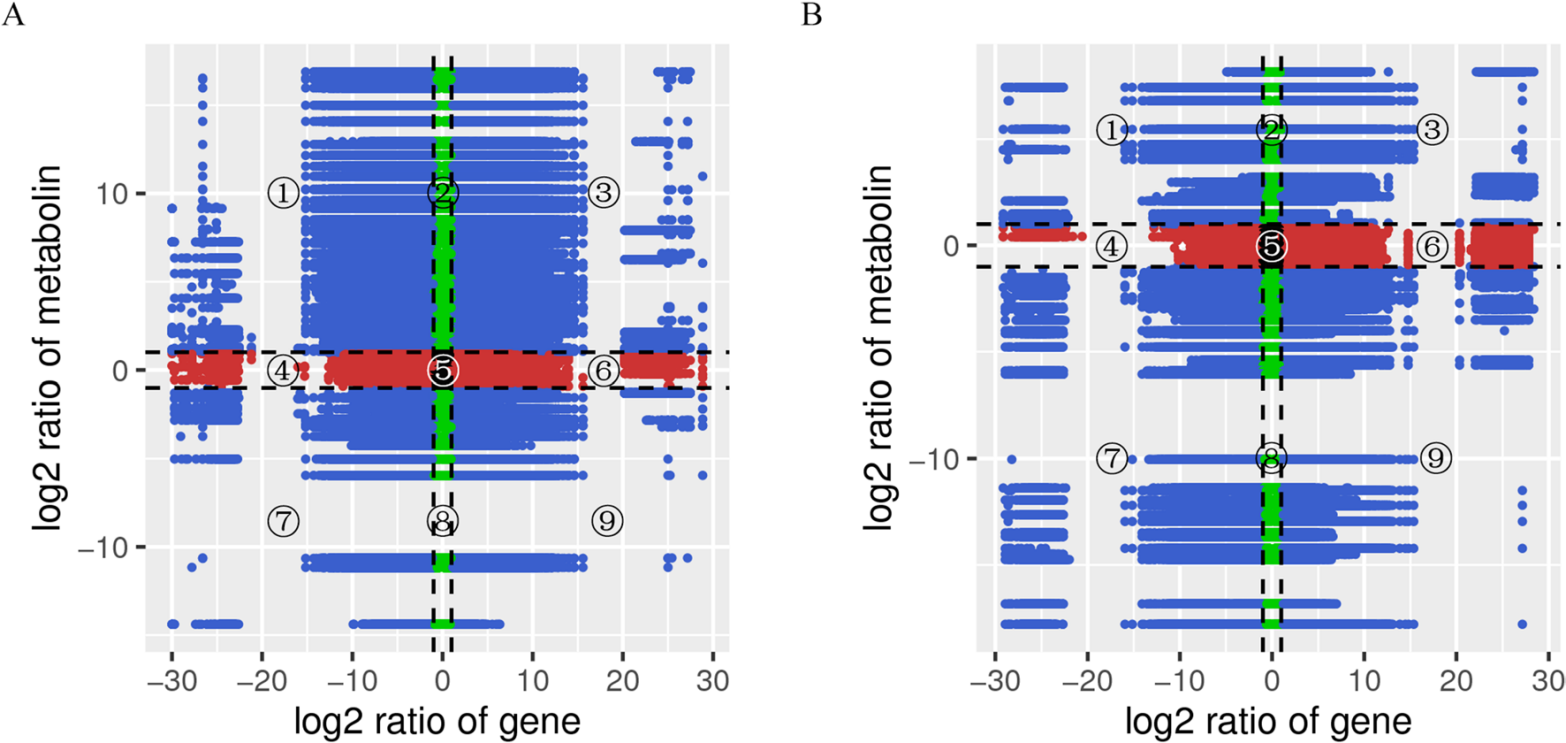
Quadrant diagrams representing the association of the DAMs and DEGs be-tween large flower taxa and small flower taxa in *Herba Epimedii*. The x-axis represents that the log2 ratio of gene and the y-axis represents the log2 ratio of metabolite; black dot-ted lines represent the different threshold; each point represents a gene or metabolite; black dots represent the unchanged genes or metabolites; green dots represent differentially ac-cumulated metabolites with unchanged genes; red dots represent differentially expressed genes with unchanged metabolites; blue dots represent both differentially expressed genes and differentially accumulated metabolites; it is divided into ①–⑨ quadrants from left to right and from top to bottom with black dotted lines; the ①, ②and ④ quadrants indicate that the expression abundance of metabolites is higher than that of genes; the ③ and ⑦ quadrants indicate that the expression patterns of genes are consistent with the metabolites; the ⑤ quadrant indicates that the genes and metabolites are not differentially expressed; the ⑥, ⑧ and ⑨ quadrants indicate that the expression abundance of metabolites is lower than that of genes. (**A)** In flowers. (**B)** In leaves.

**Figure 6:**
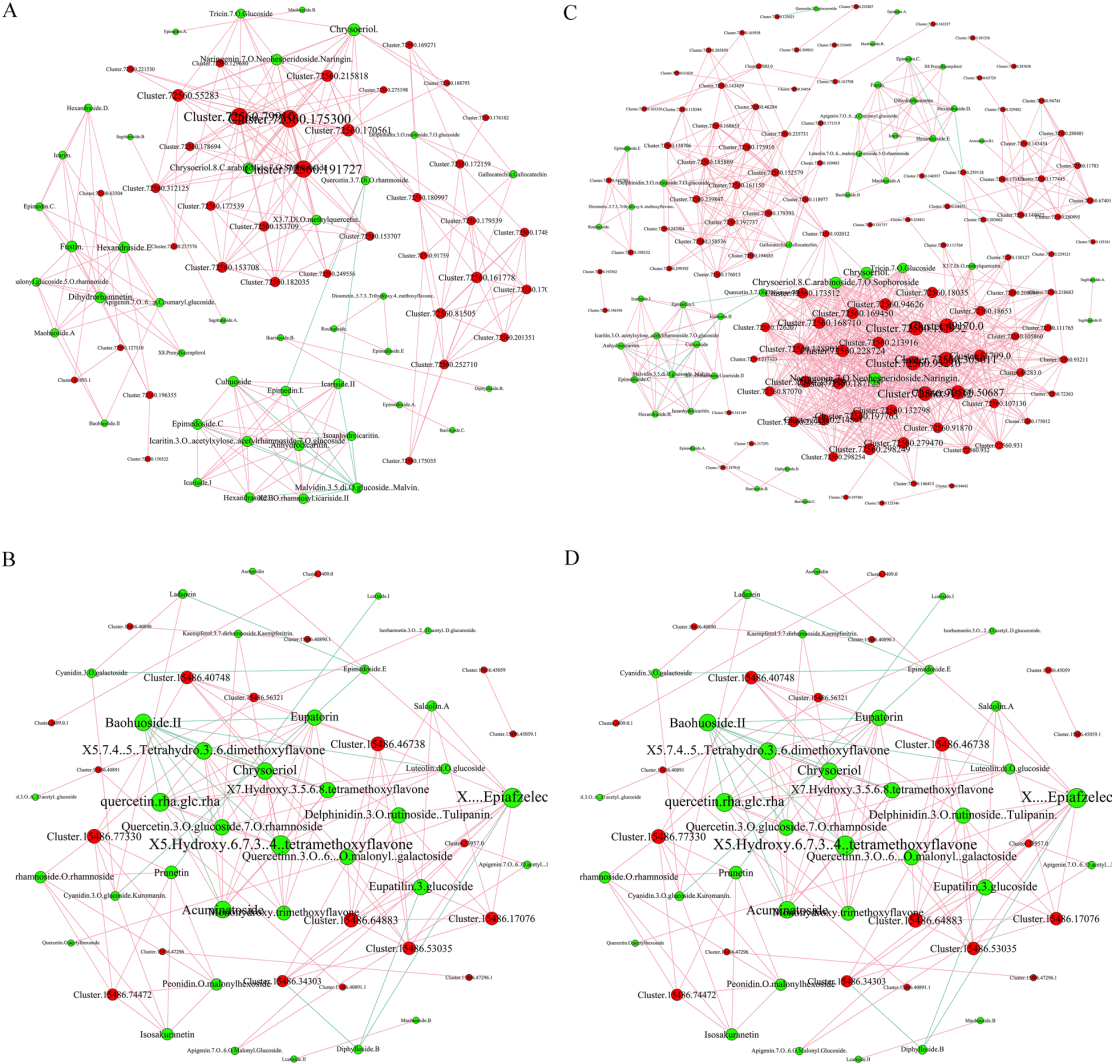
Connection network between flavonoid-biosynthesis or *O*-methyltransferase-related DEGs and flavonoid DAMs. Red nodes represent genes, green nodes represent metabolites. The size of node represents the number of related genes or metabolites. Red lines represents positive correlation, green lines represents negative correlation. **(A)** Connection network between DEGs encoding flavonoids biosynthetic enzymes and DAMs in flower. (**B)** Connection network between DEGs encoding flavonoids biosynthetic enzymes and DAMs in leaves. (**C)** Connection network between DEGs encoding O-methyltransferase and DAMs in flower. (**D)** Connection network between DEGs encoding O-methyltransferase and DAMs in leaves.

Correlation analysis was next performed on the DAMs and DEGs. Variations in the metabolites and their corresponding genes (Pearson correlation coefficient ≥ 0.8) were selected to construct nine quadrant diagrams and correlation coefficient cluster heat maps. In flowers, larger numbers of DAMs and DEGs were present in the first and third quadrants, with a negative correlation in the first quadrant and a positive correlation in the third quadrant (Fig. 5A). In leaves, a higher number of DAMs and DEGs were present in the seventh and ninth quadrants, which showed a positive correlation in the seventh quadrant and a negative correlation in the ninth quadrant (Fig. 5B). To understand the regulatory network of flavonoid biosynthesis in *Herba Epimedii*, a network analysis of DEGs encoding biosynthetic enzymes and DAMs was conducted in flower (Fig. 6A) and leaves (Fig. 6B). Based on the result of DEGs and DAMs, which were both enriched in flavonoid biosynthesis pathway, 22 structural genes showed higher correlation coefficient values (R > 0.9) with 10 flavonoids in flower, while 9 structural genes showed higher correlation coefficient values (R > 0.9) with 12 flavonoids in leaves (Supplementary Table S6). The 22 structural genes contain 1 PAL, 8 4CL, 1 CHS, 6 CHI, 4FLS in flower, and the 9 structural genes contain 1 PAL, 3 4CL, 2 CHS, 1 CHI, 2 F3’H, which are the major structural genes in the flavonoid biosynthesis pathway. Furthermore, a specific analysis of the correlation tests between these DEGs encoding *O*-methyltransferase and DAMs was also performed. The results showed that 52 and 20 DEGs encoding *O*-methyltransferase displayed a better correlation with 15 and 17 flavonoids (r > 0.9) in flower and leaves (Fig. 6C, D and Supplementary Table S6), respectively. Notably, a cluster of DEGs encoding tRNA guanosine-2'-*O*-methyltransferase, including Cluster.49170.0, Cluster.72560.305011, which is vital regulator of plant flavone and flavonol biosynthesis is closely positively related to many flavonoids (Supplementary Table S6).

### Dynamic variations in flavonoid DAMs

**Figure 7:**
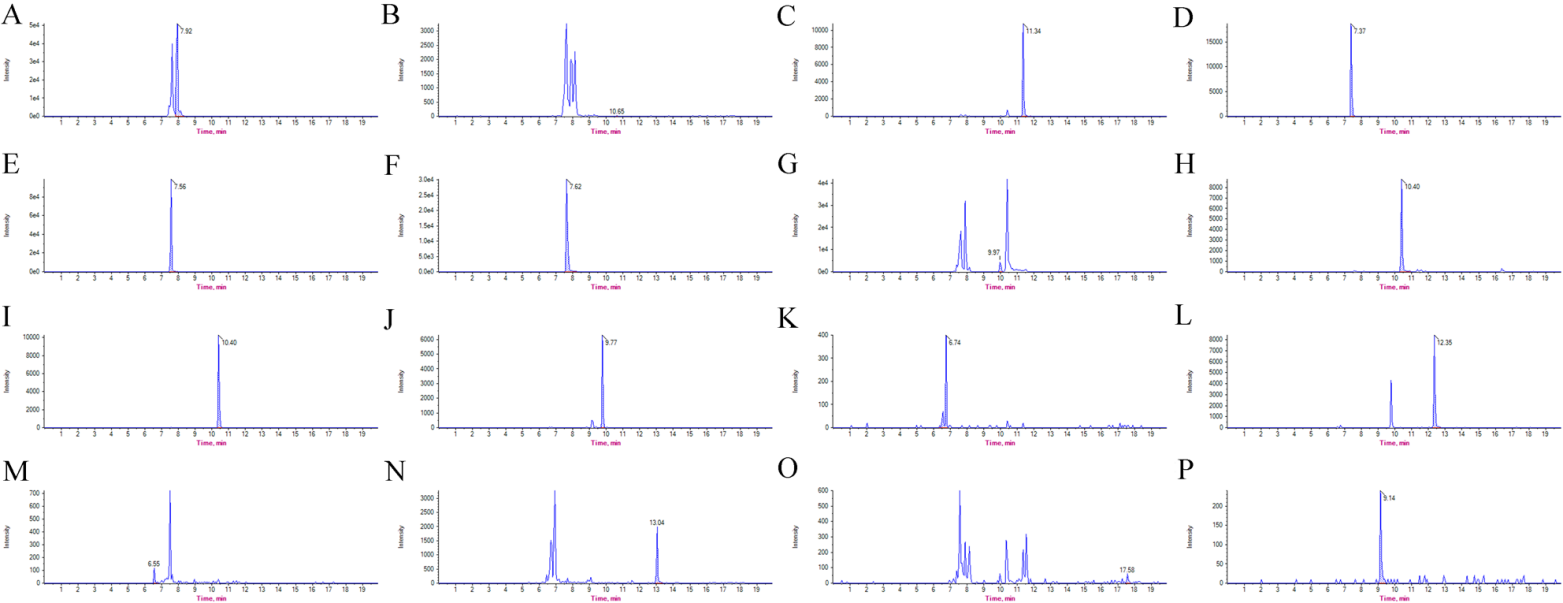
TIC diagrams of 16 flavonoid standards in the positive ion mode. **(A)** Icariin (retention time = 7.92 min). (**B)** Icariside I (retention time = 10.65 min). (**C)** Icariside II (retention time = 11.34 min). (**D)** Epimedin A (retention time = 7.37 min). (**E)** Epimedin B (retention time = 7.56 min). (**F)** Epimedin C (retention time = 7.62 min). (**G)** Sagittatoside A (retention time = 9.97 min). (**H)** Sagittatoside B (retention time = 10.40 min). (**I)** 2''-*O*-rhamnosyl icariside II (retention time = 10.40 min). (**J)** Baohuoside II (retention time = 9.77 min). (**K)** Epimedoside A (retention time = 6.74 min). (**L)** Desmethylicaritin (retention time = 12.35 min). (**M)** Baohuoside V (retention time = 6.55 min). (**N)** Acuminatin (retention time = 13.04 min). (**O)** Anhydroicaritin (retention time = 17.58 min). (**P)** Maohuoside A (retention time = 9.14 min).

To verify the content of the 16 flavonoid DAMs in large and small flower taxa, including 4 large and 4 small flower taxa cultivars (S1: *Epimedium glandulosopilosum* H.R. Liang, S2: *Epimedium leptorrhizum* Steam, S3: *Epimedium sutchuenense* Franch, S4: *Epimedium franchetii* Steam) and 4 small flower taxa cultivars (S5: *Epimedium dewuense* S. Z. He, S6: *Epimedium borealiguizhouense* S. Z. He et Y. K. Ying, S7: *Epimedium dolichostemon* Stearn, S8: *Epimedium myrianthum* Stearn), extracts were analyzed using HPLC–MS/MS. Figure 7 shows the TIC diagrams of 16 standards in the positive ion mode. Method validation including linearity, limit of detection (LOD), limit of quantification (LOQ), stability, precision, and reproducibility are shown in Table 2, with an R^2^ higher than 0.99916, relative standard deviations (RSDs) of the stability lower than 1.5%, RSDs of the precision ranging from 0.34 to 1.52%, and RSDs of repeatability in the range of 0.74–1.76%. These data highlight the reliability of the method.

**Table 2:** Method validation results including linearity, stability, precision repeatability and recovery.

No.	Name	Linearity			Stability	Precision	Repeatability	Recovery
		Regression equations	R^2^	Ranges (ng/mL)	(RSD, %)	(RSD, %)	(RSD, %)	(RSD, %)
1	Icariin	Y = 2084.18472X + 3984.49821	0.99946	1.3–13,000	< 1.5%	0.34%	0.83%	0.78%
2	Icariside I	Y = 6183.01884X + 243,873	0.99991	1.7–17,000	< 1.5%	0.56%	0.93%	0.87%
3	Icariside II	Y = 1348.23497X + 2488.12847	0.99917	1.3–13,000	< 1.5%	0.73%	1.29%	0.97%
4	Epimedin A	Y = 248.23847X + 148.14876	0.9994	1.5–15,000	< 1.5%	0.87%	0.76%	0.93%
5	Epimedin B	Y = 319.92384X + 8103.23494	0.99983	1.7–17,000	< 1.5%	0.49%	0.89%	1.08%
6	Epimedin C	Y = 243.01284X + 5193.02487	0.99928	1.4–14,000	< 1.5%	1.22%	1.27%	1.21%
7	Sagittatoside A	Y = 137.2349X + 813.02381	0.99939	2.1–21,000	< 1.5%	0.70%	1.37%	1.09%
8	Sagittatoside B	Y = 172.32498X + 2347.23847	0.99993	1.5–15,000	< 1.5%	0.92%	1.08%	0.57%
9	2''-*O*-rhamnosyl icariside II	Y = 118.34984X + 732.24791	0.99993	1.4–14,000	< 1.5%	1.32%	1.76%	0.77%
10	Baohuoside II	Y = 823.1937X + 12,438.33476	0.99948	1.6–16,000	< 1.5%	1.02%	0.76%	1.28%
11	Epimedoside A	Y = 12,487.23487X + 3298.2987	0.99992	1.1–11,000	< 1.5%	1.03%	0.99%	1.07%
12	Desmethylicaritin	Y = 1924.28773X + 12,348.4298	0.99916	1.2–12,000	< 1.5%	0.85%	0.85%	1.09%
13	Anhydroicaritin	Y = 2980.42487X + 72,787	0.99992	1.5–15,000	< 1.5%	0.94%	0.74%	0.57%
14	Maohuoside A	Y = 3882.38742X − 4912.59276	0.99973	1.6– 16,000	< 1.5%	0.96%	0.93%	0.62%
15	Baohuoside V	Y = 188.34587X − 334.08272	0.99989	1.8–18,000	< 1.5%	1.01%	0.84%	0.97%
16	Acuminatin	Y = 1847.14767X + 31,768.42986	0.99994	1.2–12,000	< 1.5%	1.52%	0.81%	0.78%

**Figure 8:**
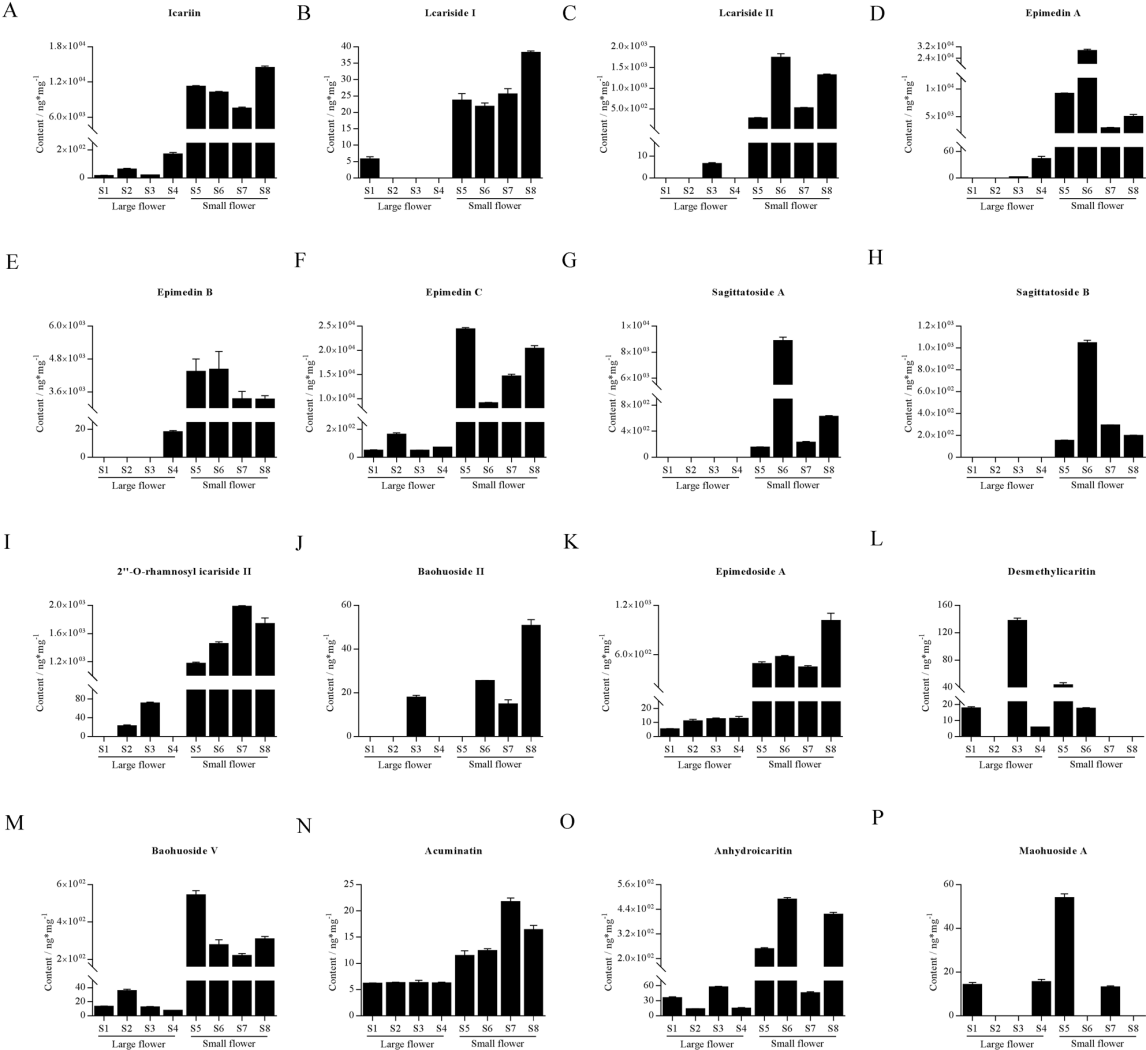
Content of 16 flavonoids between large and small flower taxa of *Herba Epimedii*, including four large and four small flower taxa cultivars. (S1: *Epimedium glandulosopilosum* H.R. Liang, S2: *Epimedium leptorrhizum* Steam, S3: *Epimedium sutchuenense* Franch, S4: *Epimedium franchetii* Steam) and four small flower taxa (S5: *Epimedium dewuense* S. Z. He, S6: *Epimedium borealiguizhouense* S. Z. He et Y. K. Ying, S7: *Epimedium dolichostemon* Stearn, S8: *Epimedium myrianthum* Stearn). (**A)** Icariin. (**B)** Icariside I. (**C)** Icar-iside II. (**D)** Epimedin A. (**E)** Epimedin B. (**F)** Epimedin C. (**G)** Sagittatoside A. (**H)** Sagittatoside B. (**I)** 2''-*O*-rhamnosyl icariside II. (**J)** Baohuoside II. (**K)** Epimedoside A. (**L)** Desmethylicaritin. (**M)** Baohuoside V. (**N)** Acuminatin. (**O)** Anhydroicaritin. (**P)** Maohu-oside A. All samples information is listed in Supplementary Table S4.

Flavonoid accumulation in the aerial regions of *Herba Epimedii* showed significant differences between large and small flower taxa. Compared with the four large flower taxa cultivars (S1, S2, S3, S4), remarkably higher levels of Icariin, Icariside I, Icariside II, Epimedin A, Epimedin B, Epimedin C, Sagittatoside A, Sagittatoside B, 2''-*O*-rhamnosyl icariside II, Epimedoside A, Baohuoside V and Acuminatin accumulated in the four small flower taxa cultivars (S5, S6, S7, S8). Baohuoside II was absent in the small flower taxa excluding S3. Interestingly, the content of Desmethylicaritin was higher than the other seven cultivars. The content of Anhydroicaritin in S7 was minimal in the small flower taxa cultivars. The content of Maohuoside A showed no significant differences between large and small flower taxa. Detailed data are shown in Fig. 8. These results suggest that the flower taxa could significantly influence the accumulation of different flavonoids in *Herba Epimedii*. The content of flavonoid DAMs was greater in the small flower taxa compared to the large flower taxa. Only four flavonoid DAMs did not match this trend.

### Expression of flavonoid biosynthetic genes

**Figure 9:**
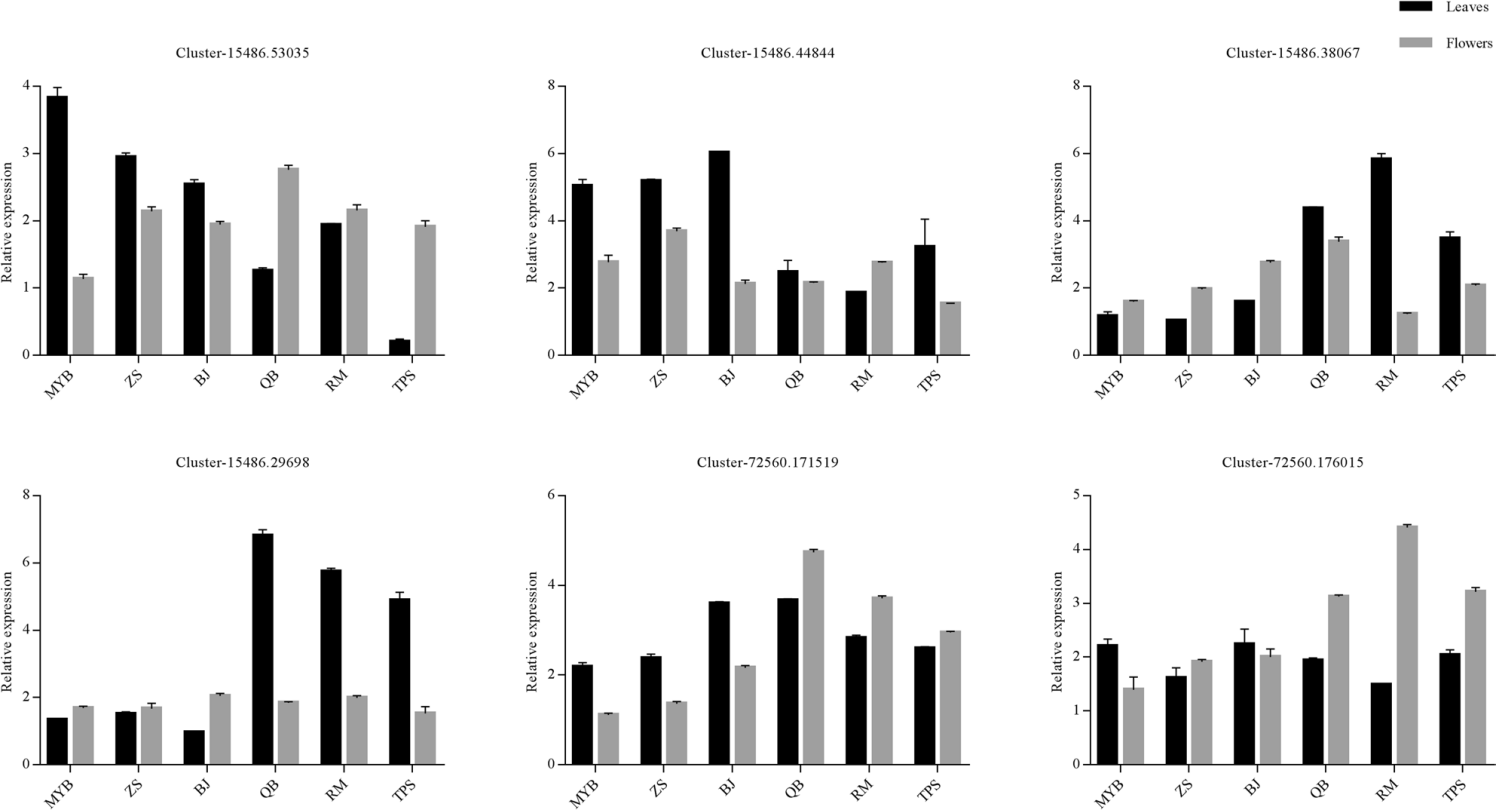
Expression levels of flavonoid biosynthetic genes between large and small flower taxa of *Herba Epimedii*. Cluster-xxxxx.xxxxxx represents the ID of gene. The x-axis indicates the relative expression level of the genes. Each value is the mean of three replicates, and error bars indicate standard deviations. All samples information is listed in Supplementary Table S4.

**Figure 10:**
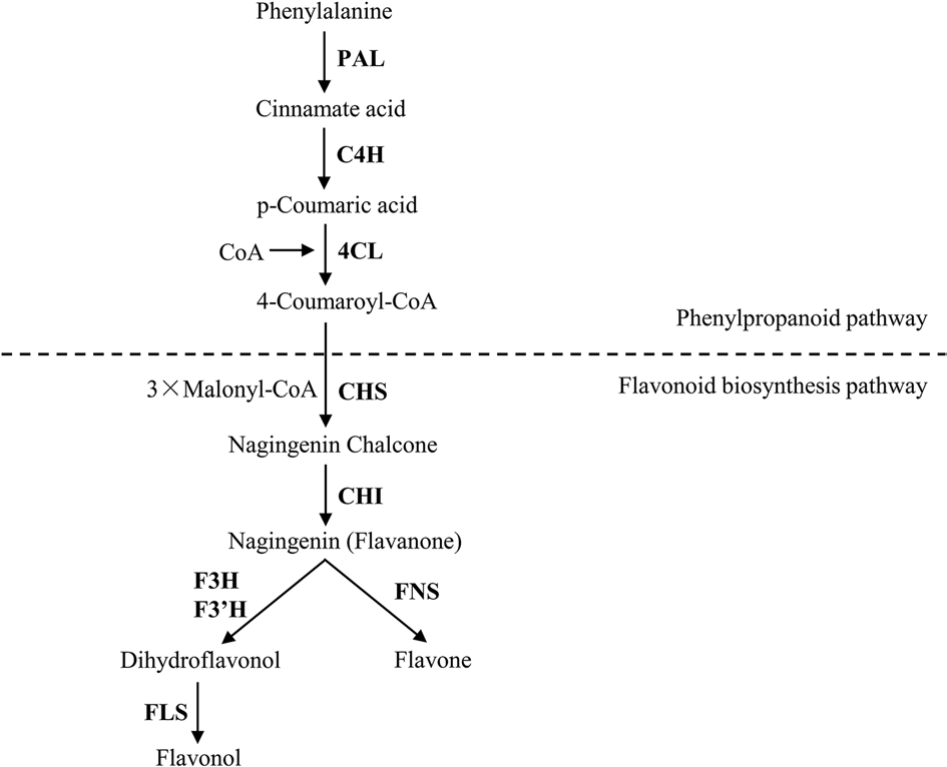
The flavonoid biosynthetic pathway in *Herba Epimedii*. Bold words indicate the key enzymes in flavonoid biosynthesis.

qRT-PCR was used to verify the relative expression of the six key enzymes in leaves and flowers involved in caffeoyl-CoA-O-methyltransferase and flavonoid biosynthesis (Fig. 9). The metabolic pathways of two common flavonoids in *Herba Epimedii* are shown in Fig. 10. The results showed that the expression of CHS (Cluster-15486.53035) and ANR (Cluster-15486.44844) were significantly downregulated in leaves, whilst the expression of DFR (Cluster-15486.38067) was upregulated in small vs. large flower taxa. Moreover, genes (Cluster-15486.29698) encoding caffeoyl-CoA-O-methyltransferase (CCOAOMT) were strikingly upregulated in the leaves of small flower taxa, and two genes (Cluster-72560.171519, Cluster-72560.176015) encoding CCOAOMT were upregulated in the flowers of the small flower taxa. These data highlight the ability of the flower taxa to induce flavonoid synthesis related genes to varying degrees, particularly those regulating the expression of CCOAOMT.

## Discussion

Previous studies have analyzed critical metabolic genes in medicinal plants using metabolic and transcriptomic analysis of different organs^9,11–15^. Flavonoids are a major source of the pharmacological activities of medicinal plants and regulate several important biological processes. A total of 10 transcription factor-encoding genes and 14 flavonoid biosynthesis genes were identified in *A. roxburghii*^25^. However, very little is known about flavonoid composition and the molecular mechanism of flavonoid biosynthesis in *Herba Epimedii*. Previous studies showed that the total flavonoid content of small flower taxa group was much higher than that of large flower taxa group using ultra performance liquid chromatography/tandem mass spectrometry (UPLC-MS/MS) such as Epimedium A, Epimedium B and Epimedium C (Fig. 8). Preliminary identification of the candidate genes involved in flavonoid accumulation were performed through comparison of the groups and organs.

Transcriptome libraries of the flowers and leaves of different groups of *Epimedium* were constructed using high-throughput sequencing technologies to identify active pharmaceutical compounds and their biosynthetic mechanisms. Flowers and leaf flavonoid metabolites in *Epimedium* were identified by UPLC-MS/MS. Once sequences were assembled, 36, 293 and 18, 505 differentially regulated genes were identified in the flowers and leaves, respectively. We identified 143 flavonoid metabolites in *Epimedium* using metabolomics. These transcriptomic and metabolomic data provide an important basis for the further exploration of metabolic pathways in *Epimedium*. Flavonoids are considered one of the major pharmaceutical components of *Epimedium*. According to KEGG analysis, flavonoid metabolites that differentially accumulate in the different organs of *Epimedium* include flavonols, flavonoid, flavanols, dihydroflavone, isoflavones, dihydroflavonol, flavonoid carbonoside anthocyanins, tannin, proanthocyanidins, chalcones and sinensetin biosynthetic pathways, which were significantly enriched. This is important information required to elucidate the molecular regulatory mechanisms underlying flavonoid accumulation.

Metabolomic analysis of the flowers revealed 43 flavonoid metabolites with greater levels in the small flower group, including Epimedin A, Epimedin B, Epimedin C, sagittatoside A, magittatoside B, maohuoside A, maohuoside B, icariside I, icariside II and icariin. Moreover, 10 flavonoid metabolites with a higher content in small flower leaves were identified, including maohuoside B, lcariside II, Epimedin B, isorhamnetin 3-*O*-β-(2''-*O*-acetyl-β-d-glucuronide), baohuoside II, Lcariside I, Epimedoside E, kaempferol 3-*O*-(6''-*O*-acetyl)-glucoside, maohuoside A and diphylloside B. In recent years, icariin has been shown to exhibit numerous pharmacological activities including anti-oxidant, anti-inflammatory, and anti-apoptotic effects. These may contribute to the described preventive and/or therapeutic benefits of icariin in various disorders in the nervous system, including cerebral ischemia, Alzheimer’s disease (AD), Parkinson’s disease (PD), multiple sclerosis (MS), and depression^26–29^. Several flavonoids including epimedin A, epimedin B and epimedin C were isolated from *Herba Epimedii*, and showed significant effects on neurite outgrowth in cultured rat pheochromocytoma (PC12h) cells^30^. In general, the leaves of *Herba Epimedii* are known to possess medicinal benefits. Nonetheless, analysis of the metabolites in the leaves of the two groups demonstrated that metabolites involved in flavonoid biosynthesis exhibit differential accumulation patterns, suggesting that suitable manipulation of the different flower groups can lead to improved flavonoid use and efficacy. These findings contribute to the evaluation of medicinal plants and increase our understanding of the applications of different *Herba Epimedii* groups.

A lack of genetic data has delayed the investigation of metabolic pathways in *Herba Epimedii*. To further elucidate the regulation of flavonoid accumulation in the different groups of this species, DEGs in leaves and flowers of the different groups identified by transcriptome sequencing were subjected to KEGG pathway enrichment analysis. The results showed the enrichment of flavonoid biosynthesis, flavone and flavonol biosynthesis, isoflavonoid biosynthesis, and upstream phenylpropanoid biosynthesis pathways in all samples. The basic metabolic pathways of flavonoids in plants have been elucidated with genes such as CHS, CHI, CYP75A, ANR, FLS and DFR, known to play a key role in the synthesis of bioactive components in medicinal plants^31–39^. In this study, the expression of FLS and DFR were upregulated in the leaves and flowers of the small flower group compared to the large flower group, which led to the enhanced accumulation of maohuoside B, lcariside II, Epimedin B, maohuoside A and diphylloside B. The results of UPLC-MS/MS were also confirmed (Fig. 8). FLS (Cluster-72560.79910), CHI (Cluster-72560.175300) and 4CL (Cluster-72560.191727) plays a significant role in regulatory network in flower, while CHS (Cluster-15486.46738), F3’H (Cluster-15486.64883) and 4CL (Cluster-15486.77330) functioned as key regulator in flavonoids metabolism.in leaves. The result is similar to the previous reports and further confirms the metabolite profiling result that the flavonoid accumulation changes in *Herba Epimedii*. O-methyltransferase plays vital roles in flavonoids metabolism, such as *C. acuminata* 10-hydroxycamptothecin O-methyltransferase (Ca10OMT)^40^ and flavonol 3-*O*-methyltransferase^41^. In this study, 52 and 20 DEGs encoding O-methyltransferase associated with flavonoid biosynthesis were identified by KEGG enrichment analysis and gene functional annotation in flower and leaves, respectively, had strongly positive correlation with flavonoids metabolites, for example, Chrysoeriol, 3,7-Di-*O*-methylquercetin and Tricin-7-*O*-Glucoside in flower, Quercetin-*O*-rhamnoside-*O*-rhamnoside, quercetinn 3-*O*-(6’’-*O*-malonyl)-galactoside and peonidin *O*-malonylhexoside in leaves. We found that DEGs encoding *O*-methyltransferase were closely to anthocyanins metabolites, such as peonidin *O*-malonylhexoside and delphinidin 3-*O*-rutinoside (Tulipanin), which might be helpful to study of flavonoids metabolism in *Herba Epimedii*.

Flavonoids are a class of 2-phenylchroman (flavan) derivatives with hydroxyl, methoxy, or hydroxy isoprene as substituents. They are methylated or glycosylated on the C4, C3 and C7 hydroxyl of the parent nucleus. This study focused on the regulatory networks of methylation in Epimedii and compared differences in *O*-methyltransferase expression between the leaves and flowers of the two groups. According to the results, 129 differentially expressed *O*-methyltransferases were observed between the leaves and flowers of large and small groups, which could be classified into 21 families. Differential expression of these *O*-methyltransferases is of particular importance to our understanding of the mechanisms underlying the metabolic differences between small and large flowers of *Herba Epimedii*.

## Conclusions

In our study, we found that the total flavonoids content was remarkably more in small flower taxa than large flower taxa of *Herba Epimedii*. The transcriptome and metabolomics responses of *Herba Epimedii* were evaluated between large flower taxa and small flower taxa, it provides much needed information on the factors that dictate flavonoid accumulation and its related genes. Small flower groups possess higher levels of flavonoids due to the upregulation of key flavonoid associated genes (CHS, ANR, DFR, and CCOAOMT) in *Herba Epimedii*. These data provide new insight when screening the planting resources of the *Herba Epimedii*.

## Supplementary Information

Supplementary Figure S1.

Supplementary Figure S2.

Supplementary Table S1.

Supplementary Table S2.

Supplementary Table S3.

Supplementary Table S4.

Supplementary Table S5.

Supplementary Table S6.
