# Supplementary figures and images for: Transcriptomic and metabolomic analysis reveals the difference between large and small flower taxa of Herba Epimedii during flavonoid accumulation
Source: Sci Rep. 2022 Feb 17;12:2762. doi: 10.1038/s41598-022-06761-z (PMC8854644; doi:10.1038/s41598-022-06761-z)

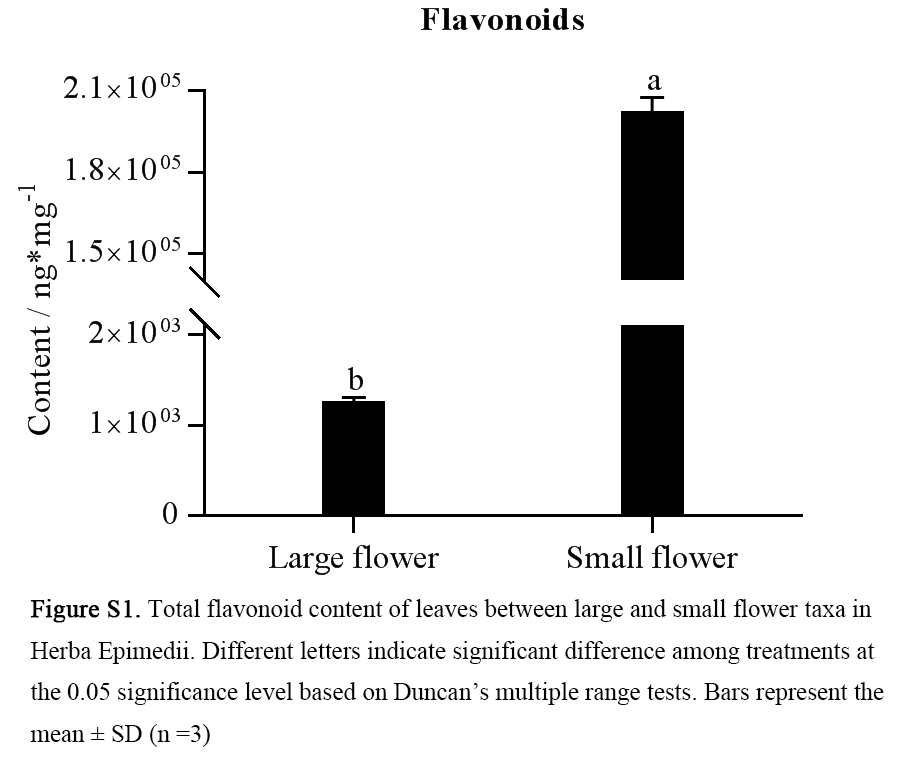

Supplement: Supplementary file 1 — Supplementary Figure S1. [file 41598_2022_6761_MOESM1_ESM.tif]

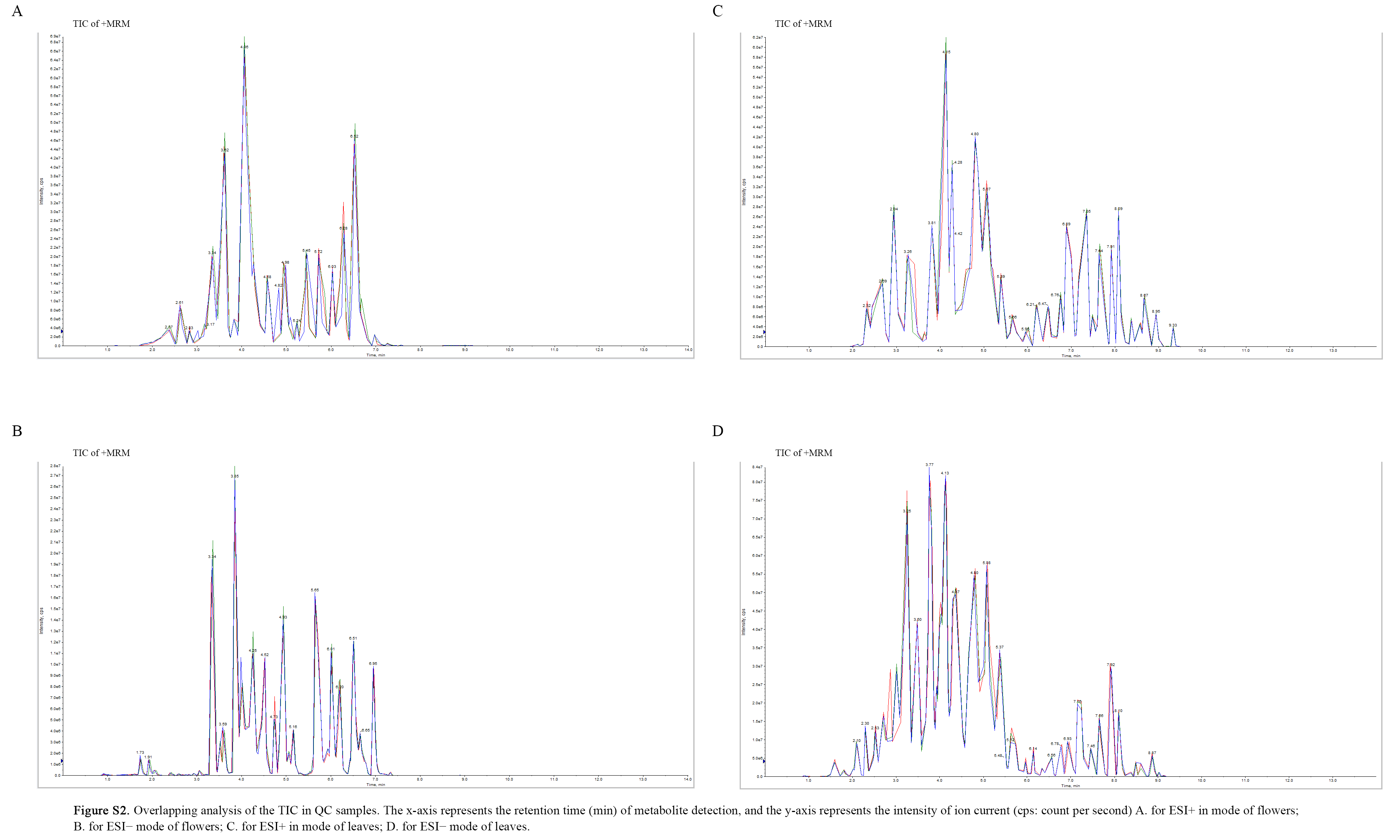

Supplement: Supplementary file 2 — Supplementary Figure S2. [file 41598_2022_6761_MOESM2_ESM.tif]
